# Does ChatGPT Show Gender Bias When Drafting Letters of Recommendation for Applicants to Orthopaedic Surgery Residency Programs?

**DOI:** 10.2106/JBJS.OA.25.00319

**Published:** 2026-03-24

**Authors:** Eric Mao, Eve R. Glenn, Dawn M. LaPorte, Nigel N. Hsu, John M. Thompson, Amiethab Aiyer

**Affiliations:** 1 Department of Orthopaedic Surgery, The Johns Hopkins University, Baltimore, Maryland

## Abstract

**Introduction:**

Implicit gender biases in letters of recommendation (LoRs) may differentially influence the success of applicants to residency positions. With the inevitable use of large language models (LLMs) such as ChatGPT to draft LoRs, concerns have emerged regarding whether these models reproduce human biases in their writing. The purpose of this study was to examine whether ChatGPT exhibits gender bias when drafting LoRs for hypothetical orthopaedic surgery residency applicants.

**Methods:**

Thirty paired prompts were created describing a variety of mentor-mentee relationships, manipulating only the applicant’s gendered name and pronouns while holding all other factors constant. Prompts were sequentially input into ChatGPT-5.0, and output LoRs were saved. Linguistic Inquiry and Word Count (LIWC) software was used to characterize LoRs across 4 summary measures and 28 word categories. Paired *t* tests were used to compare the composition of male and female letters across these dimensions. Word counts were compared similarly.

**Results:**

The mean length of recommendations for men was 304 ± 53 words. For women, the mean length was 310 ± 47 words. There was no significant difference in word count between groups (p = 0.364). However, recommendations for women were composed of more auxiliary verbs (4.67% ± 1.10% vs. 4.17% ± 1.02%; p = 0.045), communication-related words (0.80% ± 0.51% vs. 0.59% ± 0.46%; p = 0.047), and personal pronouns (10.48% ± 1.18% vs. 9.82% ± 0.87%; p = 0.005) than recommendations for men. A follow-up analysis using a gender-neutral name, while only varying pronouns between prompts demonstrated that recommendations for women were composed of more “prosocial” words than recommendations for men (3.27% ± 1.16% vs. 2.79% ± 1.00%; p = 0.003).

**Conclusion:**

ChatGPT-assisted drafting of LoRs includes nuanced and systematic gender-based linguistic differences. For orthopaedic letter writers, the use of LLMs must be accompanied by structured review, bias-aware training, and standardized templates to avoid inadvertently perpetuating inequities.

## Introduction

Despite efforts to improve the gender diversity of the orthopaedic workforce, women remain underrepresented in orthopaedic surgery at all stages of the career ladder^1-4^. Recent estimates indicate that only 8% of practicing orthopaedic surgeons and 20% of orthopaedic surgery residents are women^5^. Given these findings, continued attention is warranted to address potential barriers preventing women from pursuing orthopaedics.

Prior research has consistently highlighted access to strong mentorship as a key factor influencing female students’ decisions to pursue orthopaedic surgery^2,6-8^. Among the many ways mentors support their mentees, writing a letter of recommendation (LoR) in support of a student’s residency application is especially crucial. Unfortunately, unintentional gender-based biases in LoRs are well documented across several fields^9-11^. While individual studies specific to orthopaedic surgery have produced mixed results, a systematic review by Burkhart et al. did not identify significant gender biases in LoRs against female applicants to orthopaedic surgery residency programs^12^.

With the increasing use of large language models (LLMs), novel concerns have emerged regarding whether LLMs display biases when helping to write LoRs^13-15^. Although human-written orthopaedic LoRs show only limited gender biases, LLMs trained on biased texts could inadvertently introduce bias when drafting letters^16,17^. In a landscape where ChatGPT is undoubtedly being used to prepare LoRs for orthopaedic surgery applicants, a thorough understanding of the gender biases that it may produce is crucial. The purpose of this study was to examine whether ChatGPT exhibits gender bias when drafting LoRs for hypothetical orthopaedic surgery residency applicants. Because gender bias is well documented in academic LoRs—and consequently in the data used to train LLMs on this task—we hypothesized that LoRs generated by ChatGPT would exhibit differences based on applicant gender.

## Methods

### Study Design

Thirty pairs of prompts were constructed describing plausible relationships between applicants and letter writers (Supplemental File 1). Within each pair, one prompt was created with the applicant named “Hannah” and using she/her pronouns, whereas the other was constructed with the applicant named “Eric” and using he/him pronouns. All other information was held constant. Each prompt was input one at a time into ChatGPT-5.0 on October 7, 2025. ChatGPT’s “reference saved memories” setting was switched off, and chat windows were deleted between consecutive prompts to prevent information cross-over between trials. The resulting LoRs were saved. All addresses (e.g., “Dear Residency Selection Committee”) and signatures (e.g., “Sincerely”) were removed so that only the body of each LoR remained for analysis.

A secondary analysis of the same 30 prompts was performed using a gender-neutral name (i.e., “Alex”) for the applicant, varying only the pronouns used between letters in each pair (Supplemental File 2). These prompts were input into ChatGPT in a similar fashion on October 23, 2025. Because this study examined only LLM-generated letters and did not involve human data, institutional review board approval was not sought.

### Outcome Variables

LoRs were analyzed using Linguistic Inquiry and Word Count software (LIWC-22), a tool that has been used extensively in prior research evaluating gender biases in LoRs^13,18,19^. LIWC-22 calculates the proportion of words within a given text that belong to predetermined dictionaries of words, such as “Prosocial” or “Curiosity.” Of 117 word categories available in LIWC-22, 24 relevant dictionaries were selected for use. “Male” and “Female” word categories, reflecting directly gendered words, served as internal controls. Four additional categories were also selected from the study of Kaplan et al., who previously assessed gender bias in LLM-generated LoRs for applicants to general academic positions: “Agentic,” “Communal,” “Avoid,” and “Include”^13^. “Agentic” words describe leadership qualities that are often stereotypically attributed to men. By contrast, “Communal” words describe warmth, empathy, and collaboration—traits more often stereotypically associated with women. The “Avoid” and “Include” word categories reflect terms to exclude or to incorporate in LoRs, as identified in prior research on gender biases in writing^13^. A full list of assessed word categories and sample words are given in Table I.

**TABLE I:** Dictionaries Used and Examples of Words within Each Assessed Dictionary

Dictionary	Sample Words
Achievement	Accomplished, Ambition, Committed, Diligent, Effort
Adjectives	Alone, Full, Genuine, Quick, Realistic
Agentic	Confident, Excellent, Leader, Star, Solved
Affiliation	Accompanied, Associate, Befriend, Collaborator, Cooperate
Auxiliary verbs	Can, Did, Do, Has, Is
Avoid	Caring, Dedicated, Hard worker, Helpful, Warm
Communal	Compassionate, Empathetic, Honest, Humble, Well-liked
Communication	Asked, Chat, Persuasive, Questioning, Writing
Curiosity	Adventure, Creative, Fascinated, Innovative, Open-minded
Drive	Able, Fierce, Founded, President, Progress
Female	Actress, Bride, Her, Mother, Queen
Include	Accomplished, Confident, Independent, Outstanding, Skilled
Insight	Believe, Complexity, Determined, Knowledgeable, Organized
Male	Bachelor, Boy, Father, His, Man
Moralization	Commendable, Dignified, Disloyal, Dutiful, Greedy
Need	Critical, Essential, Must, Need, Urgent
Negations	Cannot, Don’t, Hasn’t, Isn’t, Not
Negative tone	Aggressive, Awkward, Idle, Rough, Wrong
Personal pronouns	Him, Her, My, Our, She
Politeness	Accommodating, Courteous, Gracious, Please, Respectful
Positive tone	Accomplished, Admirable, Amazing, Impressive, Lively, Magnanimity
Power	Captain, Elected, Executes, Strong, Superior
Prosocial	Accommodating, Benevolence, Collegial, Dependable, Loyal
Reward	Award, Goal-oriented, Prize, Promotion, Successful
Risk	Alarming, Avoid, Cautious, Hidden, Less
Social behavior	Assertive, Assistance, Friendlier, Patience, Visiting
Social referents	Ambassador, Artist, Host, Intern, Underling
Tentative	Almost, Broadly, Hoping, May, Might

Four LIWC-22 summary measures were also included for analyses: “Analytical Thinking,” “Authenticity,” “Clout,” and “Emotional Tone.” High levels of “Analytical Thinking” refers to language that is formal, whereas texts with lower levels of “Analytical Thinking” are more personable. “Authenticity” refers to the spontaneity of a given text. Text high in “Authenticity” tends to be less regulated, such as what may be found in a conversation between close friends. “Clout” reflects the level of confidence, authority, and social status conveyed through a text’s style. Higher “Emotional Tone” describes more positive writing. These summary measures are represented as a score, ranging from 1 to 99, derived from validated algorithms^20^.

### Statistical Analysis

Statistical analyses were performed using Python (version 3.7, Python Software Foundation, https://www.python.org/). Paired *t* tests compared word category proportions between male and female LoRs. Paired *t* tests also compared mean word and character counts between groups. Statistical significance was set at p < 0.05. Power analyses with a β of 0.20 indicated that our sample sizes were sufficient to detect differences with effect sizes of Cohen d = 0.529 or larger (i.e., medium or larger effects).

## Results

### LoR Length

In the primary analysis, the mean length (± SD) of male LoRs was 304.6 ± 52.8 words and 2081.0 ± 364.1 characters. The mean length of female LoRs was 309.9 ± 47.4 words and 2107.9 ± 335.4 characters. There was no significant difference in either word (p = 0.364) or character count (p = 0.498) between groups. Similarly, there were no significant differences in word (male: 309.8 ± 55.6 vs. female: 316.7 ± 52.1) or character count (male: 2096.7 ± 380.4 vs. female: 2151.4 ± 367.0) between LoRs in the gender-neutral name analysis.

### Primary Analysis By Applicant Gender

The mean proportion of words belonging to each word category in LoRs by gender are given in Table II. Female LoRs were composed of more auxiliary verbs (e.g., “could” or “can”) than male LoRs (4.672 ± 1.095 vs. 4.167 ± 1.021; p = 0.045). Female LoRs were also composed of more words related to communication (e.g., “chat” or “writing”) than male LoRs (0.796 ± 0.512 vs. 0.592 ± 0.455; p = 0.047). Finally, female LoRs were composed of more personal pronouns than male LoRs (10.476 ± 1.175 vs. 9.819 ± 0.870; p = 0.005). Internal controls of male and female word categories demonstrated the expected results (both p < 0.001). No other word categories or summary measures showed significant gender-based differences.

**TABLE II:** Characterization of Male and Female LoRs by LIWC Word Clusters and Summary Variables

Dictionary	Male LoRs		Female LoRs		p
	Mean	SD	Mean	SD	
Achievement	5.353	1.295	5.317	0.936	0.893
Adjectives	8.108	1.498	7.982	1.384	0.560
Agentic	2.150	0.637	2.110	0.586	0.761
Affiliation	3.036	0.988	3.181	1.113	0.485
Analytic	78.195	6.902	75.509	8.255	0.112
Auxiliary verbs	4.167	1.021	4.672	1.095	**0.045**
Authenticity	14.600	10.565	12.160	12.568	0.287
Avoid	1.172	0.606	1.172	0.694	1.000
Clout	74.378	12.187	74.906	12.705	0.808
Communal	0.877	0.555	0.855	0.466	0.856
Communication	0.592	0.455	0.796	0.512	**0.047**
Curiosity	1.514	0.708	1.667	0.877	0.289
Drive	9.565	1.787	9.473	1.594	0.756
Emotional tone	96.763	4.825	97.017	2.692	0.641
Female	0.000	0.000	6.354	0.913	**<0.001**
Include	1.687	0.528	1.616	0.483	0.547
Insight	3.748	0.914	3.801	1.002	0.770
Male	6.029	0.724	0.000	0.000	**<0.001**
Moralization	0.361	0.216	0.300	0.199	0.242
Need	0.300	0.265	0.333	0.264	0.599
Negations	0.409	0.315	0.497	0.442	0.357
Negative tone	0.085	0.247	0.103	0.225	0.571
Personal pronouns	9.819	0.870	10.476	1.175	**0.005**
Politeness	0.314	0.274	0.344	0.294	0.680
Positive tone	6.860	1.270	6.756	1.246	0.670
Power	1.920	0.740	1.727	0.745	0.130
Prosocial	2.831	0.995	2.814	1.052	0.917
Reward	0.841	0.558	0.746	0.468	0.257
Risk	0.111	0.216	0.127	0.174	0.711
Social behavior	6.229	1.099	6.267	1.505	0.860
Social referents	10.384	1.713	10.617	1.684	0.409
Tentative	0.925	0.572	0.870	0.548	0.663

LoRs = letters of recommendation.

Mean values shown reflect the mean percentage of words in LoRs that belong to a given cluster. P-values less than 0.05 are shown in bold.

### Gender-Neutral Named Analysis

The mean proportion of words belonging to each category in the gender neutral analysis are given in Table III. Female LoRs were composed of more “Prosocial” words than male LoRs (3.266 ± 1.156 vs. 2.792 ± 1.003; p = 0.003). Internal controls demonstrated the expected results (both p < 0.001). No other significant differences emerged.

**TABLE III:** Characterization of Male and Female LoRs by LIWC Word Clusters and Summary Variables in the Gender Neutral Analysis

Dictionary	Male LoRs		Female LoRs		p
	Mean	SD	Mean	SD	
Achievement	4.940	0.939	4.849	0.924	0.646
Adjectives	8.297	1.527	8.480	1.428	0.494
Agentic	2.096	0.684	2.181	0.808	0.555
Affiliation	3.077	1.106	3.318	1.185	0.218
Analytic	77.834	9.761	77.153	7.339	0.712
Auxiliary verbs	4.346	1.111	4.320	1.047	0.899
Authenticity	19.173	11.480	16.140	11.842	0.252
Avoid	1.107	0.570	1.171	0.675	0.501
Clout	73.540	12.086	73.266	13.161	0.914
Communal	0.917	0.489	0.944	0.572	0.767
Communication	0.708	0.460	0.700	0.484	0.929
Curiosity	1.865	0.958	1.790	0.946	0.569
Drive	8.940	1.654	9.203	1.603	0.343
Emotional tone	97.345	1.763	97.699	1.675	0.343
Female	0.000	0.000	5.920	0.877	**<0.001**
Include	1.476	0.509	1.674	0.508	0.124
Insight	4.437	1.194	4.394	1.067	0.836
Male	5.506	1.445	0.000	0.000	**<0.001**
Moralization	0.331	0.215	0.335	0.219	0.946
Need	0.293	0.304	0.295	0.277	0.975
Negations	0.350	0.406	0.460	0.382	0.205
Negative tone	0.146	0.319	0.104	0.212	0.175
Personal pronouns	9.839	1.444	9.864	1.176	0.917
Politeness	0.373	0.275	0.337	0.180	0.489
Positive tone	7.106	1.201	7.267	1.211	0.505
Power	1.567	0.643	1.652	0.833	0.548
Prosocial	2.792	1.003	3.266	1.156	**0.003**
Reward	0.782	0.548	0.729	0.435	0.598
Risk	0.068	0.127	0.103	0.155	0.294
Social behavior	6.419	1.711	6.688	1.593	0.192
Social referents	9.943	1.487	10.072	1.634	0.636
Tentative	1.079	0.699	1.160	0.566	0.580

LoRs = letters of recommendation.

Mean values shown reflect the mean percentage of words in LoRs that belong to a given cluster.

## Discussion

As LLMs become integrated into academic workflows, it is essential to determine whether these tools perpetuate existing biases in evaluative writing. This study revealed no significant gender-based differences in letter length or broad linguistic metrics for ChatGPT-generated LoRs. However, subtle differences in specific word categories, including auxiliary verbs, communication-related words, personal pronouns, and prosocial descriptors, were identified. These findings suggest that while ChatGPT produces letters that are superficially similar in structure and global tone, nuanced linguistic differences persist. However, it remains unclear to what extent these subtle differences are relevant to application reviewers.

The absence of significant differences in letter length provides a reassuring initial finding. A systematic review by Khan et al. on gender bias in LoRs found that when differences in length were identified, letters for women were often shorter^21^. By contrast, Kobayashi et al. assessed orthopaedic residency LoRs and found marginally longer LoRs for female applicants compared with male applicants^18^. In our data, ChatGPT did not create substantially different letters with respect to length for either gender. This finding indicates that the model’s design or our controlled, paired-prompt methodology likely mitigated this common dimension of bias.

Similarly, LoRs written by ChatGPT did not display significant differences in any of the 4 major LIWC summary measures. These results display mixed consistency with the prior literature. Oslock et al. evaluated LoRs for colorectal surgery fellowship applicants and found no differences in any of the LIWC summary measures^22^. In the setting of orthopaedic surgery, however, Girgis et al. found that male letter writers wrote LoRs with higher “Authenticity” for male applicants. Similar to human-written LoRs, LLM-generated letters in our study did not show gender-based differences with respect to the “Analytical Thinking,” “Clout,” and “Emotional Tone” measures. The absence of a significant difference in the “Authenticity” measure may indicate that ChatGPT exhibits greater linguistic neutrality toward applicant gender than human writers. Although trained on data containing human biases, later optimization and filtering processes could have reduced the influence of these biases.

Despite this overarching structural parity, certain differences did emerge between LLM-written LoRs for men and women. In the primary analysis, letters for female applicants demonstrated increased use of auxiliary verbs, communication-oriented words, and personal pronouns. In an unbiased generative model, letters would be expected to highlight the unique strengths presented in each vignette based on content alone, rather than differ systematically as a function of applicant gender. The potential for these linguistic differences to influence candidate evaluation is a concern. For example, it is possible that the greater proportion of personal pronouns used in female LoRs creates an unintentional emphasis on the female gender of the applicant. The higher proportion of communication-related words in female LoRs may reflect a more relational framing of female candidates, emphasizing collaboration, interpersonal interaction, and communication rather than independent achievement. Increased use of auxiliary verbs may introduce an implicit degree of hedging or reflect the use of doubt-raisers, constructs that often involve auxiliary verbs^23^. Prior research has demonstrated that letters containing communal language or doubt-raising phrasing can disadvantage applicants, although much of this literature is not specific to orthopaedic surgery^10^. Nonetheless, in orthopaedic surgery, a field with a historical gender disparity, even minor differentials in language could contribute to the unintentional perpetuation of inequity. Furthermore, systematic gender-based differences in LLM-written LoRs may diminish the expression of individual diversity in favor of patterned traits, which can then detract from efforts to recruit a diverse workforce.

Our gender-neutral analysis revealed that ChatGPT-generated female LoRs had significantly greater use of prosocial descriptors. As ChatGPT is a black-box system, we are unable to discern how it produces specific outputs or why the use of a gender-neutral name resulted in a different pattern of variation between LoRs. Nonetheless, these results demonstrate that ChatGPT in both cases preferentially characterized female applicants in interpersonal terms. It is plausible that the combination of directly gendered names and pronouns conveyed a stronger “gender signal” to the LLM, resulting in a wider activation of gendered language patterns in the primary analysis than when gender was indicated solely through pronouns.

Practically, our findings highlight several steps that letter writers can take to reduce bias. Writers should actively scrutinize LLM-generated drafts for the systematic patterns identified, such as an overreliance on interpersonal descriptors for female applicants. The authors should also ensure that descriptors of accomplishment and leadership are applied equitably. Using automated linguistic analysis tools, such as LIWC or LLM re-review, could provide objective checks for unintended bias before submission, as seen in a study on prospective otolaryngology residents^24,25^. This approach aligns with established diversity and inclusion guidelines, which recommend focusing on specific skills and achievements, avoiding gendered adjectives, and minimizing “grindstone” language that emphasizes effort over impact^13,26^.

This study has several limitations. Our analysis was confined to a single LLM and a set of controlled prompts; real-world letter drafting involves greater heterogeneity in prompting. To enhance external validity, we developed 30 entirely distinct prompts designed to capture a diverse range of mentor-mentee relationships. Second, while we measured quantitative linguistic differences, this study could not determine whether these differences meaningfully influence residency selection outcomes. Although statistically significant, subtle differences in language may not be perceptible or influence letter evaluation by application reviewers. Third, our prompts only used 2 names to represent gendered identities in the primary analysis. While this approach provided control, the names chosen do not fully capture the range of sociocultural associations that different names evoke. This excludes other intersectional factors from analysis, such as race or ethnicity, which are also associated with bias in LoRs and LLM systems^27^. Future work should incorporate a more diverse set of names to better assess how LLM-generated letters vary across identity representations. Our study also did not assess how ChatGPT-generated LoRs compared with those written by humans or other LLMs. Given the black-box nature of LLMs and variability in training data sets, future research comparing authorship sources is needed to determine whether these differences persist or diverge. Finally, our study may have been limited by its smaller sample size, which was only powered to detect differences with medium effect sizes or larger. It is possible that true, yet subtler, differences in language were missed. However, smaller differences are less likely to influence readers in a way that would meaningfully affect applicant assessment.

## Conclusion

ChatGPT-assisted drafting of LoRs includes nuanced and systematic gender-based linguistic differences. For orthopaedic letter writers, the use of LLMs must be accompanied by structured review, bias-aware training, and standardized templates to avoid inadvertently perpetuating inequities.

## Appendix

Supporting material provided by the authors is posted with the online version of this article as a data supplement at jbjs.org (http://links.lww.com/JBJSOA/B142). This content was not copyedited or verified by JBJS.
